# Estimation of the Number of Scans Required per Hard-to-Clean Location and Establishing the Limit of Quantification of a Partial Least Squares Calibration Model When the FTIR Is Used for Pharmaceutical Cleaning Verification

**DOI:** 10.3390/molecules27144569

**Published:** 2022-07-18

**Authors:** Apu Sarwar, Conor McSweeney, Mark Smith, Eric Moore

**Affiliations:** 1 Pfizer Ireland Pharmaceuticals, Ringaskiddy, P43 X336 Cork, Ireland; apu.sarwar@pfizer.com (A.S.); conor.mcsweeney@pfizer.com (C.M.); mark.r.smith2@pfizer.com (M.S.); 2 School of Chemistry, University College Cork, T12 K8AF Cork, Ireland

**Keywords:** pharmaceutical cleaning verification, FTIR for cleaning, sample size estimation, LOQ for FTIR, rapid cleaning verification

## Abstract

This study aims to identify two critical components required for pharmaceutical cleaning verification when an FTIR is used: (a) the number of scans required per hard-to-clean location, and (b) the limit of quantification (LOQ) of the FTIR instrument when measuring the surface contamination. The current practice in pharmaceutical manufacturing does not require multiple samples as it is standard practice to collect a single swab sample from a 25 × 25 cm area from a difficult-to-reach area of the manufacturing equipment. However, since the FTIR will only scan a tiny portion of the surface compared to the swab, a sufficient number of samples (data points) are required to provide enough confidence to ensure that the measurement results are close to the true value with a maximum degree of certainty. Similarly, calculating the LOQ for a linear regression could be straightforward. However, complexity arises when the experimental data are complex; in this case, the complexity arises due to the nature of the measurement and the lack of the defined peak in the pre-processed spectra. Therefore, this study uses the practical approach of calculating the sample size and the LOQ.

## 1. Introduction

### 1.1. Sample Size Estimation

In a traditional cleaning verification study, the swabbing of the equipment is normally performed in a hard-to-clean location. The hard-to-clean location is chosen based on the assumption that the equipment is most likely to be dirty at those locations where it is difficult to reach by hand [1]. Usually, it is a narrow area or a corner of the manufacturing equipment. In a typical swab study, a 25 cm^2^ area is swabbed using a textile swab and a suitable solvent. The specification for surface residue is expressed as µg/cm^2^, below which the surface is considered clean [2]. As new technology emerges, the swabbing practice is becoming less popular [3]. More real-time measurement is now demonstrating its capability. It is now realistic to assume that pharmaceutical cleaning verification would be a real-time analysis using FTIR or other spectroscopic technology in the near future [4,5,6,7,8,9,10,11].

The FTIR technology is capable of scanning only a few millimeter squares area in a single scan. Therefore, to get a comparable or better measurement to swab, the sample size needs to be established using a scientific and statistical justification in order to ensure the number of samples taken from a hard-to-clean location is sufficient, and that it provides enough confidence that the results obtained from the area using the FTIR are accurate and close to the true value.

A number of established approaches can be used to estimate sample size using a statistical justification. One of the most common approaches used to establish the sample size is calculating the Margin Of Error (MOE) [12]. The MOE estimates the random sampling error by approximating a parameter, such as the mean or percentage. A margin of error is typically used in survey results. The MOE can also be used to calculate the sample size before conducting a study to ensure that the number of samples collected will provide enough confidence that the error is within the acceptable boundary.

In this investigation, the MOE was calculated using the Relative Standard Deviation (RSD) of the signal from the API on the surface where the API was deposited non-uniformly. The near-to-highest RSD was used to calculate the MOE to minimize the inaccurate results. 

### 1.2. Estimation of LOQ for Spectroscopic Measurement

The objective of the validation of an analytical procedure is to demonstrate that it is suitable for its intended purpose. The analytical procedure should be clearly understood since this will govern the validation characteristics, which need to be evaluated. One of the critical components of the validation parameters is LOQ. According to ICH guidelines, the rapid cleaning validation/verification using an FTIR will fall under the category of ‘testing for impurities’. Therefore, the LOD and LOQ will be required to be calculated for this analytical method. The quantitation limit of an individual analytical procedure can be described as the lowest amount of analyte in a sample, which can be quantitatively determined with suitable precision and accuracy. The quantitation limit is a parameter of quantitative assays for low levels of compounds in sample matrices and is used particularly for the determination of impurities and/or degradation products [13,14,15].

According to the ICH guidelines [16], the following approaches are suggested for determining the detection limit and quantitation limit:

[1.] Based on Visual Evaluation
[2.] Based on Signal-to-Noise Approach
[3.] Based on the Standard Deviation of the Response and the Slope.
  - Based on the Standard Deviation of the Blank.
  - Based on the Calibration model.

For ‘Based on Visual Evaluation’, the visual evaluation is not suitable for the FTIR measurement as it is designed for non-instrument methods [17]. The Signal-to-Noise is widely used to calculate the LOQ/LOD. However, when the cleaning verification is performed using an FTIR, the spectrum will be collected from a very low surface residue concentration. The spectrum pre-processing will be needed to develop the calibration model when using FTIR for cleaning verification. Therefore, the spectrum may not have well-defined peaks in many cases. In addition, the baseline shift may occur due to the coupon-to-coupon surface roughness variation. All these factors will make it challenging to determine the LOQ/LOD using the signal-to-noise approach.

The standard deviation calculation of collected spectra from a blank coupon would require a calibration model. However, the model range may not cover the blank concentration when using the FTIR. The calibration model-based approach is not the best approach as this method is normally used for simple linear regression. In this case, the PLS model will be used, and the calculated LOQ might result in an unnecessarily higher value which may not reflect the true LOQ of the FTIR detector itself.

According to USP <1225> and European Union Reference Laboratory (EURL) technical report 2016”, the standard deviation of responses could be used to determine the LOQ and LOD. Low concentrated analyte (pseudo blank) can be used to calculate the LOQ/LOD. The standard deviation of ‘minimum 10 independent measurements at very low concentrations of analyte’ could be used to calculate the LOD and LOQ. In this investigation, this approach was examined, and it was found that the LOQ was more relevant to the capability of the FTIR as well as the analytical method [18,19].

## 2. Materials and Methods

### 2.1. Material and Instrumentation

The experiments were performed using finish# 8 stainless steel coupons or plates. These coupons were supplied by Laser Technology, Dublin, Ireland. HPLC-grade water and ethanol were purchased from Sigma-Aldrich (Wicklow, Ireland), which were used to clean the coupon and for standard preparation. The Active Pharmaceutical Ingredient (API) was provided by the Pfizer manufacturing network. The Chem-Cal microdot printer from Photon System Inc. (PSI, Covina, CA, USA) was used to deposit targeted amounts of APIs uniformly onto the surface stainless steel coupons. Gilson P200 and P1000 microliter pipettes were used to dilute the stock solutions, and a Mettler Toledo XSR105DU analytical balance was used for weighing the solid compounds. These instruments were used as per vendor guidelines.

The Agilent 4300 Hand-held Mid-IR system (Agilent Technologies, Santa Clara, CA, USA) with a specular reflectance interface was used in this study. The Mid-IR system was equipped with a 450 specular reflectance interface with a 1.76 mm^2^ spot size. The instrument was controlled using Agilent’s Micro-lab software (version V5.3.1748). The Mid-IR system was configured to collect data over a wide wavelength range (650–4000 cm^–1^). The instrument is capable of performing non-destructive testing, providing instant feedback via the onboard computer display. The Minitab^®^ 20.2 (64-bit) software was used to perform the statistical analysis.

### 2.2. Description of the Method

#### 2.2.1. Establishment of Visual Cleanliness Limit

Active Pharmaceutical Ingredients (APIs) were printed on three coupons, and the printed API was observed multiple times. The limit was established when any lower amount of API was not visible in a poorly lit area.

#### 2.2.2. Calculate Relative Standard Deviation (RSD)

API was deposited non-uniformly using a microliter syringe on the coupon, and the coupon was scanned using FTIR 4300. The RSD was calculated for each coupon from the collected spectra. The RSD was calculated using all the collected spectra (minimum of 25–30 spectra were collected) from each cupon. Additionally, each of those spectra represents 16 individual scans. Hence, each of the collected spectrums is an average of 16 FTIR scans. The number ‘16’ was chosen carefully as a higher number of the spectrum will improve the spectral resolution but will take longer. Since this is a hand-held FTIR and the scan will be taken from the surface while holding the FTIR using a single hand, the number of scans was carefully selected to ensure that the spectrum is of reasonable quality. At the same time, collecting the spectrum does not take a very long time.

A pre-build calibration model was used to predict the concentration for each collected spectra, and the predicted results were used to calculate the RSD. The calibration model was not developed based on the entire range of the FTIR spectrum. Instead, the model was based on the area of interest where the strong peaks were present. For example, the model range for API A was 1200.20–1259.84 cm^–1^, and for API B, the ranges were 1187.15–1289.65 cm^−1^ and 2884.96–2993.05 cm^–1^. The details of how the calibration model is developed can be found in the previous publication [2].

#### 2.2.3. Applicability of the Sample Size

In order to assess the applicability of the approach described in this investigation, two APIs were investigated. In each case, API was deposited inhomogeneously onto three stainless steel coupons, giving an approximate average surface residue of 0.6 µg/cm^2^. The APIs were printed on the coupons using the Chem-Cal printer. The Chemical printer deposits up to 5200 microdroplets on the surface of the coupon with a drop size of 4–10 nL, and the distance between the droplets is 0.5–1 mm. More detail on the API deposition techniques is described in the previous publication [2]. The coupons were visually assessed, and in each case, the residue was visible. A total of 10 measurements were taken at random locations on each coupon using the hand-held FTIR instrument. For each API, a pre-developed calibration model was used to predict the surface residue of the individual measurements. An average of the 10 individual measurements were calculated for each coupon, representing the overall surface concentration predicted by the FTIR with a reduced sample size of 10. This experiment was performed in triplicate for each API, and the final results are tabulated in results section.

#### 2.2.4. Determination of the LOQ

A coupon was printed with the low targeted amount of API. Ten individual spectra were collected from a fixed point without moving the FTIR data collection interface and the sample.

## 3. Results

### 3.1. Estimation of Visual Limit and Typical Surface Residue Variability

Based on laboratory studies for several products, a suitable value corresponding to visual cleanliness was established as 0.6 µg/cm^2^. Therefore, the limit at which residue could not be visually detected for those products was <0.6 µg/cm^2^. All the cleaning verification measurements must be performed only if the visual inspection is passed. Therefore, all the experiments in this investigation were performed at the visual limit. One other critical component of the cleaning verification is the Residue Acceptance Limit (RAL). In this investigation, the RAL is assumed to be 1 µg/cm^2^ or greater. For the majority of products, the RAL will typically be >1 µg/cm^2^. The value used in this experiment is to simulate a worse case. However, where the RAL for a specific product is <1 µg/cm^2^, further assessment will be required, and the sample size should be estimated on a case-by-case basis.

During laboratory studies, different APIs were assessed. In each case, the API was inhomogeneously deposited onto a stainless-steel coupon by hand in order to simulate a highly variable distribution of residue across the surface. The %RSD of these coupons ranged from 27–53% RSD. Therefore, the selection of 50% RSD here was considered suitable, as a potential worst case, with the actual %RSD of residue on surface equipment (post-cleaning) expected to be significantly lower.

To allow a reliable mean of the surface residue to be calculated from a number of points randomly measured across the surface, there must either be:

- A low variability across the surface, requiring only a limited number of points to be measured, or
- A larger number of points to be measured where there is high surface variability.

### 3.2. Approach for Estimating Sample Size/Number of Samples

**Figure 1:**
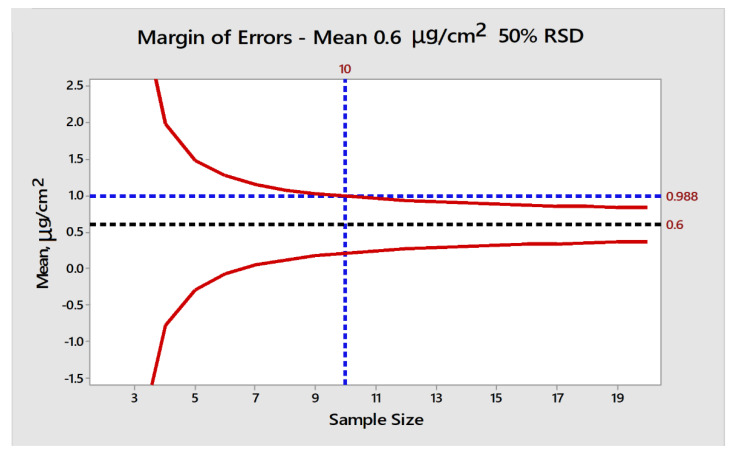
Calculated MOE for each sample size.

The MOE was established by considering the worst-case scenario and because the measurement would only be performed after the successful visual inspection. The MOE was calculated at a 99% confidence interval, and the standard deviation was estimated as 0.3 µg/cm^2^ (i.e., 50% RSD of the example visual cleanliness limit (Table 1)). Based on the number of points measured/averaged (i.e., the *x*-axis), the estimate of the mean may be between the red lines (representing ±3σ of the mean), with the true mean located at 0.6 µg/cm^2^ (Figure 1).

The method is a good approximation of potential error around the actual mean. It is 0.6 µg/cm^2^, or the visual limit in this case. However, a more accurate assessment would be based on non-parametric estimates, i.e., the mean value cannot go below zero. Therefore, a 3σ limit is used to estimate a worse case and support the subsequent estimation of method capability.

In this scenario, the upper range of the mean (+3σ of the mean), based on randomly measuring 10 samples across a surface with a %RSD of 50%, would be 0.988 µg/cm^2^. This approach, based on measuring 10 sample points across a surface of a hard-to-clean location of the manufacturing equipment, minimizes the risk of potentially reporting a mean above the RAL that is actually lower than the RAL (i.e., a false positive). Similarly, the visual inspection prevents any reporting of the false-negative result as any residue above 0.6 µg/cm^2^ would be traceable during the visual inspection.

The capability of this approach was further evaluated using the method capability CpK index (Table 2). The method capability (Cpk) was estimated based on the number of samples and subsequent Margin of Error Figure 1.

$$\mathrm{CpK}=\frac{\mathrm{USL}-\mathrm{Mean}}{3\sigma }$$

USL = Upper specification limit, in this case, the residual acceptance limit (i.e., μg/cm^2^)

Mean = Historical Mean/Visual Limit (i.e., 0.6 μg/cm^2^)

σ = Margin of Error (calculated for each sample size)

The method capability measures how good the method is (risk of failure as a result of the method variability based on the specification limit).

**Figure 2:**
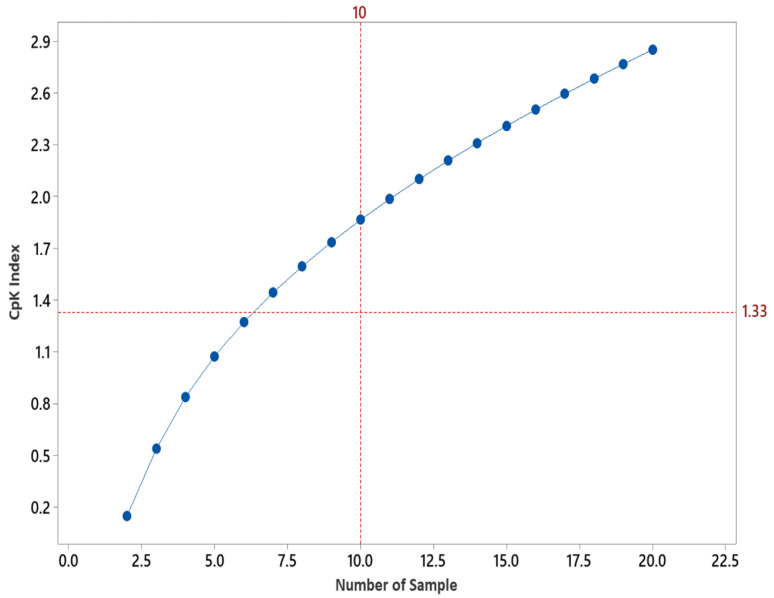
The graph shows the CPK index against the sample size.

According to the CpK index value in Table 2 and the measured CpK index value in Figure 2, to achieve a method capability of 1.33 or more, in this example, would re-quire a minimum of seven samples across the surface of the hard-to-clean location (based on the specification limit and estimate of variability). Due to the potential un-der-estimation of the Margin of Error (based on the use of parametric statistics), it is recommended that a method capability of 1.67 is set (i.e., a minimum of 10 samples across the surface would give a CpK index greater than 1.67.

### 3.3. Sample Size Application:

In order to assess the applicability of the approach described in the section above, two APIs were investigated. In each case, the API was deposited inhomogeneously onto three stainless steel coupons (by hand using a Hamilton syringe) to give an approximate average surface residue of 0.6 µg/cm^2^. The coupons were visually assessed, and in each case, the residue was visible.

A total of 10 measurements were taken at random locations on each coupon using the hand-held Rapid Cleaning Verification (RCV) instrument. For each API, a pre-developed calibration model was used to predict the surface residue of the individual measurements, with an average calculated for each coupon (Table 3).

The average surface residue of the 10 individual measurements did not exceed the range of 0.212–0.988 µg/cm^2^ for any API. This represents the average surface residue range based on the approach described in this investigation (i.e., based on an average of 0.6 µg/cm^2^ and 50% RSD surface residue variability). The applicability of the approach for measuring 10 samples was demonstrated across two different APIs where the RAL is more than 1 µg/cm^2^.

### 3.4. Limit of Quantitation (LOQ)

The 10 measurements were collected from the same location of the coupon without moving the instruments of the coupon. At the same time, the detector temperature was monitored to ensure that the detector temperature was not changed. The data was collected with a new background.

The standard deviation of the ten measurements was calculated, and the LOQ was calculated using the formula outlined above. The LOQ for the molecule analyzed was 0.56 µg/cm^2^, which indicates that the calibration model must not develop for this molecule below the value of 0.56 µg/cm^2^. Some molecules may have higher or lower RAL. Therefore, LOQ might change for that particular molecule. It is recommended that a LOQ study is performed for each molecule separately using the standard deviation of the 10 discreet measurements collected from the same location as the pseudo blank coupon.

## 4. Conclusions

This study was performed to define an acceptable risk in the measured mean or true value (i.e., how far the current cleaning process is from the cleaning specification and therefore the required method capability). This should be assessed carefully, with relevant Quality and Operations input. Two options for selecting this value:

[1.] The surface residue is related to the limit where the surface is defined as visually clean.
  - Where the surface is identified as visually unclean, the cleaning operation would be repeated. Therefore, there should be no potential for measuring a surface above this visual limit.
[2.] The historical mean for the specific cleaning operation is being evaluated.
  - Where historical data indicates the residue after cleaning is very low (i.e., significantly below the limit for visual cleanliness), it would be acceptable to select the historical mean.

However, for a new product, a single homogeneous coupon is recommended to be prepared at 0.6 µg/cm^2^ and visually assessed. If the coupon can be identified as visually unclean, it would be appropriate to utilize the detailed approach. Where the visual limit is higher, a further assessment of the suitability of 10 points may be required. The sample size estimation approach described in this investigation would not be suitable if the visual inspection failed. In addition, during the sample size estimation assessment, the practicality of the data collection must be considered. An excessive amount of data points might improve the accuracy, but the repetitive nature of the measurement will cause more measurement error and, at a point, it would be a burden to the end-user. Therefore, the risk and the benefit must be carefully assessed. A balanced and suitable sample size should suggest that it would not cause a burden to the operator during data collection, and the measurement will provide enough confidence that the results obtained are close to the true value.

It is important to characterize the analytical performance of a laboratory test in order to understand its capability and limitations and to ensure that they are “fit for purpose”. The terms LOD and LOQ describe the smallest concentration of a measurand that an analytical procedure can reliably measure.

There are a number of different ways that the LOQ and LOD are calculated. However, the calculation of the LOQ and LOD for a Partial Least Square (PLS) model is different from the linear calibration model. Considering all suggested approaches, “standard deviation of the response of the lowest concentration of analyte” was preferred to calculate the LOQ and LOD for the FTIR during rapid cleaning verification/validation measurements. Once the LOQ/LOD is calculated, the calibration model validation can be performed. All validation coupons should be prepared above the LOQ concentration for model validation.

## Figures and Tables

**Table 1:** Parameters used to calculate the MOE.

No.	Parameters Used MOR	Value
1	RAL (uppur limit)	1 µg/cm^2^
2	Visual Limit (theoretical mean)	0.6 µg/cm^2^
3	%RSD	50
4	Standard Diviation (SD)	0.3 µg/cm^2^ (based on a coupon prepared at the visual limit of 0.6 µg/cm^2^)
5	Confidence interval (CI)	99.73% (i.e., 3σ of the mean)

**Table 2:** CPK index reference value.

CpK Index Value	Method Performance
<1.33	Poor
1.33–1.67	Acceptable
>1.67	Good
>2.00	Excellent

**Table 3:** A sample size of 10 was used to estimate the amount of API on the surface.

Name of the API/DS	RAL (µg/cm^2^)	Coupon No	API Residue Visible	Average Surface Residue on Each Coupon (µg/cm^2^)
API 1	2.00	1	Yes	0.550
		2	Yes	0.482
		3	Yes	0.816
API 2	5.04	1	Yes	0.400
		2	Yes	0.627
		3	Yes	0.636

## Data Availability

Data used in this article are available from the authors.

## Sample Availability

Samples of the compounds are not available from the authors.
